# Four‐year incidence of major adverse cardiovascular events in patients with atherosclerosis and atrial fibrillation

**DOI:** 10.1002/clc.23344

**Published:** 2020-02-27

**Authors:** Benjamin Miao, Adrian V. Hernandez, Yuani M. Roman, Mark J. Alberts, Craig I. Coleman, William L. Baker

**Affiliations:** ^1^ University of Connecticut School of Pharmacy Storrs Connecticut USA; ^2^ Hartford Hospital Hartford Connecticut USA; ^3^ Vicerrectorado de Investigacion Universidad San Ignacio de Loyola (USIL) Lima Peru; ^4^ Neuroscience Institute Hartford Hospital Hartford Connecticut USA

**Keywords:** atrial fibrillation, established atherosclerotic disease, major adverse cardiovascular events

## Abstract

**Background:**

There is a paucity of contemporary data assessing the implications of atrial fibrillation (AF) on major adverse cardiovascular events (MACE) in patients with or at high‐risk for atherosclerotic disease managed in routine practice.

**Hypothesis:**

We sought to evaluate the 4‐year incidence of MACE in patients with or at risk of atherosclerotic disease in the presence of AF.

**Methods:**

Using US MarketScan data, we identified AF patients ≥45 years old with billing codes indicating established coronary artery disease, cerebrovascular disease, or peripheral artery disease or the presence of ≥3 risk factors for atherosclerotic disease from January 1, 2013 to December 31, 2013 with a minimum of 4‐years of available follow‐up. We calculated the 4‐year incidence of MACE (cardiovascular death or hospitalization with a primary billing code for myocardial infarction or ischemic stroke). Patients were further stratified by CHA_2_DS_2_‐VASc score and oral anticoagulation (OAC) use at baseline.

**Results:**

We identified 625,951 patients with 4‐years of follow‐up, of which 77,752 (12.4%) had comorbid AF. The median (25%, 75% range) CHA_2_DS_2_‐VASc score was 4 (3, 5) and 64% of patients received an OAC at baseline. The incidence of MACE increased as CHA_2_DS_2_‐VASc scores increased (*P*‐interaction<.0001 for all). AF patients receiving an OAC were less likely to experience MACE (8.9% vs 11.6%, *P* < .0001) including ischemic stroke (5.4% vs 6.7%, *P* < .0001).

**Conclusion:**

Comorbid AF carries a substantial risk of MACE in patients with or at risk of atherosclerotic disease. MACE risk increases with higher CHA_2_DS_2_‐VASc scores and is more likely in patients without OAC.

## 1 INTRODUCTION

Atherosclerotic disease and atrial fibrillation (AF) share many of the same comorbidities and risk factors^1^ and frequently coexist. Compared to the general population, AF prevalence in patients with established atherosclerotic disease is fivefold higher (2.3% vs 11.7%, respectively).^2^ Both are independently associated with elevated major adverse cardiovascular events (MACE) risk and understanding the synergistic increases in incidence rates and comparative risk based on disease characteristics may greatly aid treatment efforts by identifying patient factors associated with high risk.^3^ The Reduction of Atherothrombosis for Continued Health (REACH) registry prospectively evaluated patients at risk for or with established atherosclerotic disease.^4^ Subanalyses of REACH showed a 4‐year MACE incidence of 24.3% in patients with comorbid AF.^3^ They also found a linear trend in the correlation between MACE and CHA_2_DS_2_‐VASc scores. However, REACH enrolled patients between 2003 and 2004 with follow‐up through 2008. Advances in the management of AF in the past decade, such as the approval of non‐vitamin K antagonist oral anticoagulants (NOAC) and their recommendation over warfarin in evidence‐based guidelines, may have lowered MACE risk.^5^, ^6^, ^7^ While the early hazard of MACE in AF patients has been previously evaluated, such studies only focused on incident AF patients and did not require patients to have atherosclerotic disease.^8^ It is therefore important to identify MACE incidence in a more contemporary population reflecting modern medical practices. This study aimed to estimate the contemporary 4‐year incidence of MACE for United States AF patients with or at risk for established atherosclerotic disease in routine practice, the association between CHA_2_DS_2_‐VASc scores and MACE risk, and how oral anticoagulation (OAC) use modifies this risk.

## 2 METHODS

We performed a retrospective claims database analysis using US IBM MarketScan data from January 1, 2013 through December 31, 2017. MarketScan combines two separate databases, a commercial and a Medicare supplemental database, to cover all age groups; and contains claims from 260 contributing employers, 40 health plans, and government and public organizations representing ~240 million lives.^9^ MarketScan captures enrollment records, demographics, International Classification of Diseases, Tenth‐Revision (ICD‐10) diagnosis codes (and cross‐walked ICD‐9 codes), procedure codes, admission and discharge dates, outpatient medical services data, and prescription dispensing records. All MarketScan data are de‐identified and are compliant with the Health Insurance Portability and Accountability Act of 1996. This study was determined by our institutional review board to not constitute research involving human subjects according to 45 Code of Federal Regulations 46.102(f) and was deemed exempt from board oversight.

We identified eligible patients according to similar criteria used for the REACH Registry by examining claims data between January 1, 2013 and December 31, 2013 (calendar year 2013). All patients ≥45‐years‐of‐age with comorbid AF (with or without valve disease) and established coronary artery disease (CAD; ie, diagnosis codes suggesting a history of stable or unstable angina, percutaneous coronary intervention, coronary artery bypass grafting, or myocardial infarction), cerebrovascular disease (CVD; ie, diagnosis codes suggesting a history of ischemic stroke or transient ischemic attack) or peripheral artery disease (PAD; ie, diagnosis codes suggesting a history of peripheral arterial disease or a prior intervention including angioplasty, stenting, atherectomy, peripheral arterial bypass grafting, or amputations) or with three or more risk factors for atherosclerotic disease (ie, diagnosis codes suggesting a history of diabetes, diabetic nephropathy, carotid stenosis, hypertension, hypercholesterolemia, smoking, or age ≥65 years for men or ≥70 years for women) were included. Patients were further stratified by baseline CHA_2_DS_2_‐VASc^10^ score and OAC use (apixaban, edoxaban, rivaroxaban, dabigatran, or warfarin).

Our main outcome measure was the incidence rate of MACE (composite outcome of cardiovascular mortality, myocardial infarction, or ischemic stroke). Secondary outcomes include individual MACE components. Cardiovascular mortality was defined as death occurring in‐hospital within 14 days of a myocardial infarction, ischemic stroke, heart failure, acute coronary syndrome, coronary artery bypass grafting, or percutaneous coronary intervention. Baseline data included demographics, vascular disease status, atherothrombotic risk factors, oral anticoagulants and other medications, and CHA_2_DS_2_‐VASc scores. Patient selection, baseline characteristics, and outcome measure identification were based on the presence of ICD‐10 (or cross‐walked ICD‐9) billing codes from medical and prescription claims. Starting on January 1, 2014, patients who met eligibility criteria during calendar year 2013 were followed for 4 years (patients with at least 3 years and 9 months of follow‐up were included in the analysis) or until endpoint occurrence.

Baseline characteristics were analyzed using descriptive statistics. Categorical data are reported as percentages and continuous data as medians with accompanying 25%, 75% ranges. Outcomes are reported as cumulative proportions or incidences (ie, events/patients) and incidence rates (ie, events/100 person‐years [PYs]). A multivariable Cox proportional hazard regression adjusting for known factors that influence cardiovascular outcomes (ie, age, sex, smoking, chronic kidney disease, and statin use),^11^ components of the CHA_2_DS_2_‐VASc score, medications relevant to the treatment and management of atherosclerotic disease and warfarin, NOAC or absence of OAC use was performed. The proportional hazards assumption was tested based on Schoenfeld residuals and was found to be valid for all outcomes. Associations are reported as adjusted hazard ratios (HRs) with 95% confidence intervals (CIs).The presence of statistical interactions of MACE across CHA_2_DS_2_‐VASc scores were tested using the methods described by Altman and Bland.^12^ All data management and statistical analysis was performed using IBM SPSS Statistics version 25.0 (IBM Corp., Armonk, New York) and SAS 9.4 (SAS Institute Inc., Cary, North Carolina).

## 3 RESULTS

**Figure 1:**
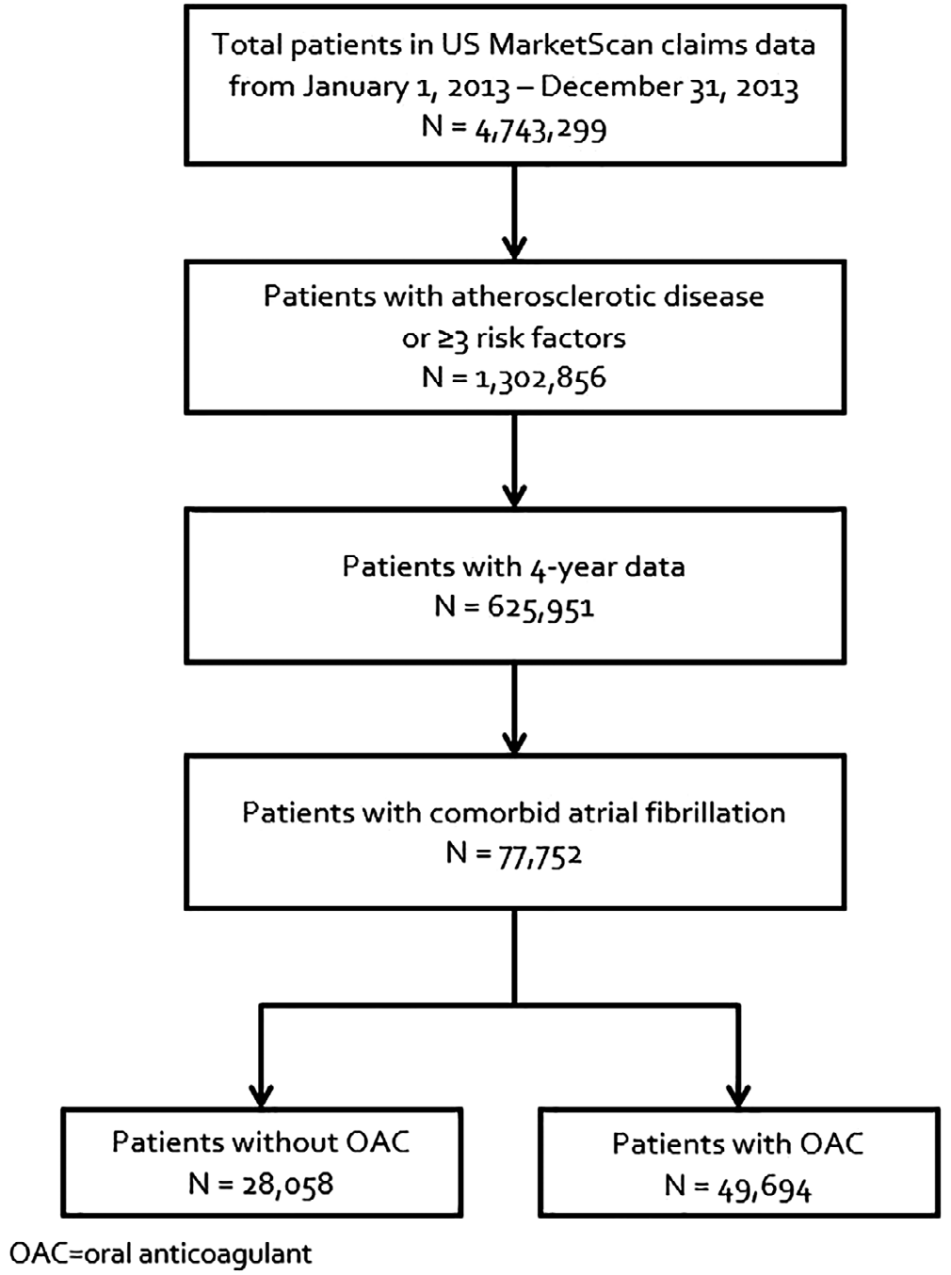
Flow diagram of patient selection

We identified 625,951 patients with established atherosclerotic disease or ≥3‐risk factors and 4‐years of follow‐up, of which 77,752 (12.4%) had comorbid AF (Figure 1). The patients with AF had a median (25%, 75% range) age of 75 (69, 81) years, CHA_2_DS_2_‐VASc score was 4 (3, 5) and 26,921 (34.6%) had valvular disease. Patients with established atherosclerotic disease accounted for 46.8% of our population; among these, 56.2% had CAD, 27.8% had CVD, 36.4% had PAD, and 18.9% had polyvascular disease. The other 53.2% of the population had only ≥3‐risk factors. Most patients had hypertension (98.5%) and hypercholesterolemia (80.3%) and just under half had diabetes. An angiotensin‐converting enzyme inhibitor or angiotensin receptor blocker was used by 63% of patients, ~76% received a statin, ~15% a P2Y12 inhibitor, 74% a beta‐blocker, ~64% an OAC, and ~24% an antiarrhythmic at baseline (Table 1).

**Table 1:** Baseline characteristics of patients with 4‐years of follow‐up

	All N = 77,752	Total established disease N = 36,398	Any coronary artery disease^a^ N = 20,468	Any cerebrovascular disease^a^ N = 10,128	Any peripheral artery disease^a^ N = 13,256	Multiple risk factors only N = 41,354
Demographics (n, %)						
Age (median, 25%, 75% range)	75 (69, 81)	75 (68, 81)	74 (67, 81)	76 (69, 82)	76 (69, 82)	76 (70, 81)
Vascular disease status (n, %)						
Coronary artery disease	20,468 (26.3)	20,468 (56.2)	20,468 (100)	2511 (24.8)	3736 (28.2)	0 (0)
Cerebrovascular disease	10,128 (13.0)	10,128 (27.8)	2511 (12.3)	10,128 (100)	1787 (13.5)	0 (0)
Peripheral artery disease	13,256 (17.0)	13,256 (36.4)	3736 (18.3)	1787 (17.6)	13,256 (100)	0 (0)
Polyvascular disease	6874 (8.8)	6874 (18.9)	5667 (27.7)	3718 (36.7)	4943 (37.3)	0 (0)
CHA_2_DS_2_‐VASc score (median, 25%, 75% range)	4 (3, 5)	4 (3, 5)	4 (3, 5)	5 (4, 6)	4 (3, 5)	4 (3, 5)
Congestive heart failure	25,863 (33.3)	14,902 (40.9)	9651 (47.2)	3769 (37.2)	5480 (41.3)	10,961 (26.5)
Hypertension	76,610 (98.5)	35,467 (97.4)	20,141 (98.4)	9787 (96.6)	12,919 (97.5)	41,143 (99.5)
Age ≥75	41,835 (53.8)	19,148 (52.6)	9988 (48.8)	5675 (56.0)	7520 (56.7)	22,687 (54.9)
Diabetes	37,114 (47.7)	15,895 (43.7)	9250 (45.2)	4174 (41.2)	6426 (48.5)	21,219 (51.3)
Prior stroke, TIA or thromboembolism	8741 (11.2)	7876 (21.6)	2126 (10.4)	7094 (70.0)	1727 (13.0)	865 (2.1)
Vascular disease (prior MI, peripheral artery disease or aortic plaque)	10,131 (13.0)	9286 (25.5)	8624 (42.1)	1517 (15.0)	2229 (16.8)	845 (2.0)
Age 65 to 74	23,589 (30.3)	10,280 (28.2)	6156 (30.1)	2703 (26.7)	3637 (27.4)	13,309 (32.2)
Female	32,047 (41.2)	14,474 (39.8)	7034 (34.4)	4884 (48.2)	5364 (40.5)	17,573 (42.5)
Risk factors (n, %)						
Diabetic nephropathy	2167 (2.8)	1008 (2.8)	607 (3.0)	267 (2.6)	421 (3.2)	1159 (2.8)
Carotid stenosis	11,215 (14.4)	6903 (19.0)	3623 (17.7)	2790 (27.5)	2882 (21.7)	4312 (10.4)
Hypercholesterolemia with treatment	62,413 (80.3)	26,804 (73.6)	16,176 (79.0)	7291 (72.0)	9289 (70.1)	35,609 (86.1)
Smoker	3828 (4.9)	1960 (5.4)	1203 (5.9)	516 (5.1)	802 (6.1)	1868 (4.5)
Age ≥70 in females or ≥65 in males	63,183 (81.3)	28,073 (77.1)	15,423 (75.4)	7941 (78.4)	10,695 (80.7)	35,110 (84.9)
Valvular disease^b^ (n, %)	26,921 (34.6)	14,695 (40.4)	9150 (44.7)	4095 (40.4)	4974 (37.5)	12,226 (29.6)
Medication use (n, %)						
Oral anticoagulants	49,694 (63.9)	22,243 (61.1)	12,065 (58.9)	6477 (64.0)	8271 (62.4)	27,451 (66.4)
Non‐vitamin K oral anticoagulants^c^	16,060 (20.7)	7286 (20.0)	4014 (19.6)	2230 (22.0)	2525 (19.0)	8774 (21.2)
Warfarin^c^	36,280 (46.7)	16,350 (44.9)	8813 (43.1)	4744 (46.8)	6243 (47.1)	19,930 (48.2)
Antiarrhythmics	18,542 (23.8)	9008 (24.7)	5979 (29.2)	2066 (20.4)	2867 (21.6)	9534 (23.1)
Amiodarone	9208 (11.8)	5199 (14.3)	3749 (18.3)	1068 (10.5)	1657 (12.5)	4009 (9.7)
Dronedarone	2467 (3.2)	1142 (3.1)	729 (3.6)	245 (2.4)	367 (2.8)	1325 (3.2)
Other antiarrhythmic agents	7917 (10.2)	3221 (8.8)	1889 (9.2)	867 (8.6)	999 (7.5)	4696 (11.4)
Digoxin	13,325 (17.1)	5959 (16.4)	3361 (16.4)	1546 (15.3)	2317 (17.5)	7366 (17.8)
ACE/ARB	48,964 (63.0)	22,576 (62.0)	13,428 (65.6)	5967 (58.9)	8072 (60.9)	26,388 (63.8)
β‐blockers	57,513 (74.0)	27,627 (75.9)	16,610 (81.2)	7271 (71.8)	9775 (73.7)	29,886 (72.3)
Calcium channel blockers	31,360 (40.3)	14,211 (39.0)	7777 (38.0)	4269 (42.2)	5307 (40.0)	17,149 (41.5)
Diuretics	44,505 (57.2)	21,118 (58.0)	12,379 (60.5)	5344 (52.8)	8122 (61.3)	23,387 (56.6)
Antidiabetic agents	24,888 (32.0)	10,523 (28.9)	6248 (30.5)	2669 (26.4)	4215 (31.8)	14,365 (34.7)
Statin	59,305 (76.3)	25,676 (70.5)	15,568 (76.1)	7023 (69.3)	8834 (66.6)	33,629 (81.3)
P2Y12 inhibitors	11,706 (15.1)	8730 (24.0)	6406 (31.3)	2155 (21.3)	2855 (21.5)	2976 (7.2)
NSAIDs including COX‐2 inhibitors	11,726 (15.1)	5510 (15.1)	3150 (15.4)	1482 (14.6)	1984 (15.0)	6216 (15.0)

Abbreviations: ACE/ARB, angiotensin‐converting enzyme inhibitor or angiotensin receptor blocker; COX‐2, cyclooxygenase‐2 enzyme inhibitors; NSAID, nonsteroidal anti‐inflammatory drugs.

a These cohorts overlap each other.

b Defined according to the Elixhauser comorbidity index.

c Groups are not mutually exclusive. Patients may have received both medications during the baseline period.

Major adverse cardiovascular events occurred in 9.9% (2.95 events/100 PYs) of patients at 4‐years, including an incidence of 11.6% (3.60 events/100 PYs) in patients with atherosclerotic disease and 8.4% (2.41 events/100 PYs) in patients with multiple risk factors only. Myocardial infarction, ischemic stroke and cardiovascular death occurred in 4.6% (1.34 events/100 PYs), 5.8% (1.71 events/100 PYs), and 1.6% (0.47 events/100 PYs) of patients, respectively. Among those with established disease, MACE incidences were the highest in patients with CVD (14.0%) followed by PAD (11.7%) and CAD (11.6%) (Table 2).

**Table 2:** Incidences and rates of cardiovascular outcomes at 4‐years

	All *N* = 77,752 events/100PY (95% CI) n (%)	Total established disease N = 36,398 events/100PY (95% CI) n (%)	Any coronary artery disease^a^ N = 20,468 events/100PY (95% CI) n (%)	Any cerebrovascular disease^a^ N = 10,128 events/100PY (95% CI) n (%)	Any peripheral artery disease^a^ N = 13,256 events/100PY (95% CI) n (%)	Multiple risk factors only N = 41,354 events/100PY (95% CI) n (%)
MACE	2.95 (2.88‐3.01)	3.60 (3.49‐3.71)	3.58 (3.43‐3.72)	4.41 (4.19‐4.65)	3.70 (3.52‐3.89)	2.41 (2.33‐2.49)
	7699 (9.9)	4239 (11.6)	2369 (11.6)	1416 (14.0)	1554 (11.7)	3460 (8.4)
Myocardial infarction^b^	1.34 (1.30‐1.38)	1.73 (1.66‐1.81)	2.07 (1.96‐2.18)	1.49 (1.37‐1.63)	1.84 (1.71‐1.97)	1.02 (0.97‐1.07)
	3562 (4.6)	2081 (5.7)	1393 (6.8)	496 (4.9)	787 (5.9)	1481 (3.6)
Ischemic stroke^b^	1.71 (1.67‐1.76)	1.98 (1.90‐2.06)	1.60 (1.50‐1.69)	3.30 (3.11‐3.50)	1.88 (1.75‐2.01)	1.49 (1.43‐1.56)
	4543 (5.8)	2380 (6.5)	1084 (5.3)	1072 (10.6)	803 (6.1)	2163 (5.2)
Cardiovascular‐related death†	0.47 (0.44‐0.49)	0.55 (0.51‐0.59)	0.51 (0.46‐0.56)	0.65 (0.57‐0.74)	0.63 (0.56‐0.71)	0.40 (0.37‐0.43)
	1257 (1.6)	673 (1.8)	350 (1.7)	221 (2.2)	276 (2.1)	584 (1.4)

a These cohorts overlap each other.

b Outcomes are not mutually exclusive.

Abbreviations: CI, confidence interval; MACE, major adverse cardiovascular events; PY, person‐years.

**Figure 2:**
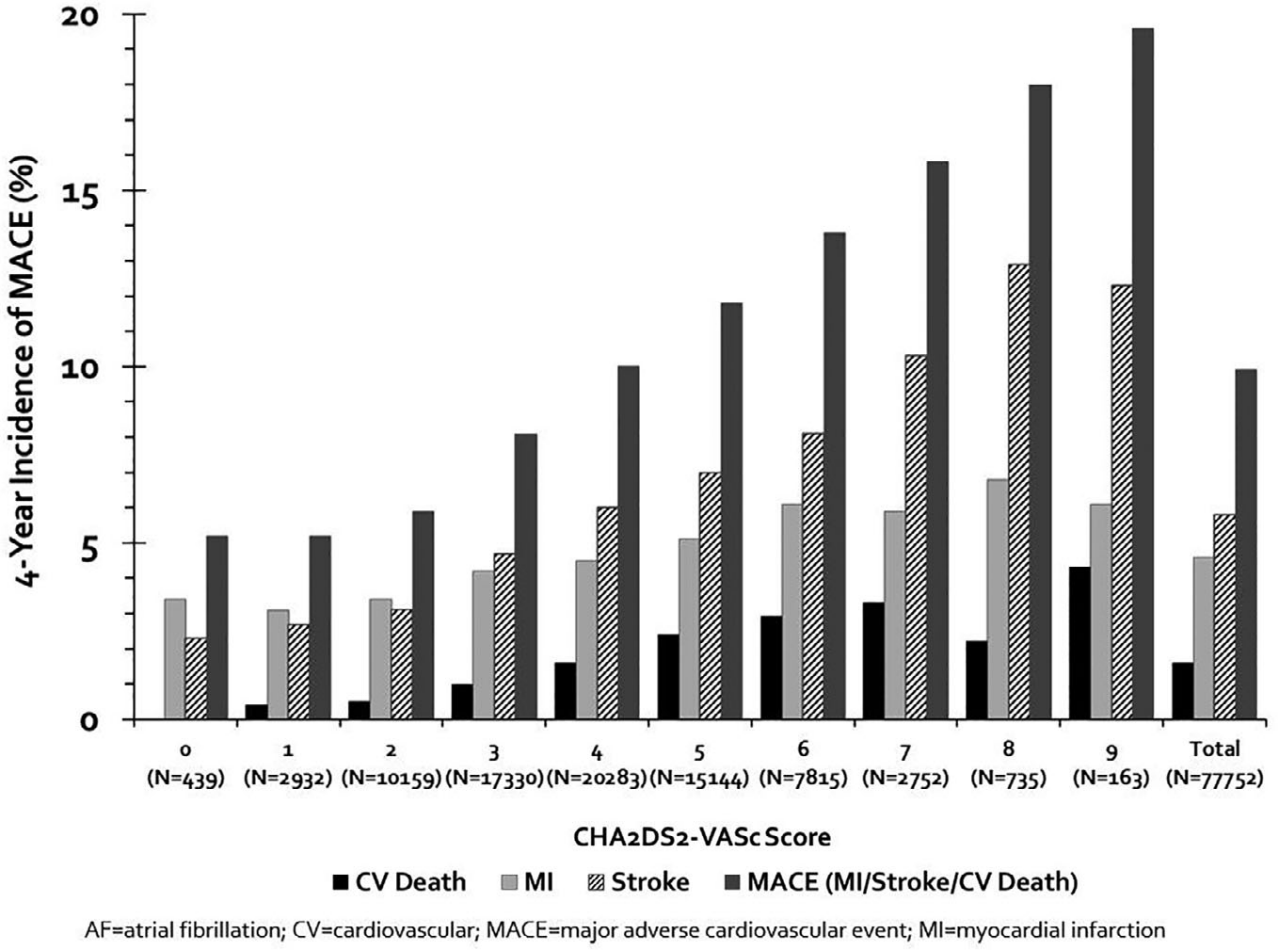
Incidence of MACE stratified by CHA2DS2‐VASc score. MACE, major adverse cardiovascular events

**Figure 3:**
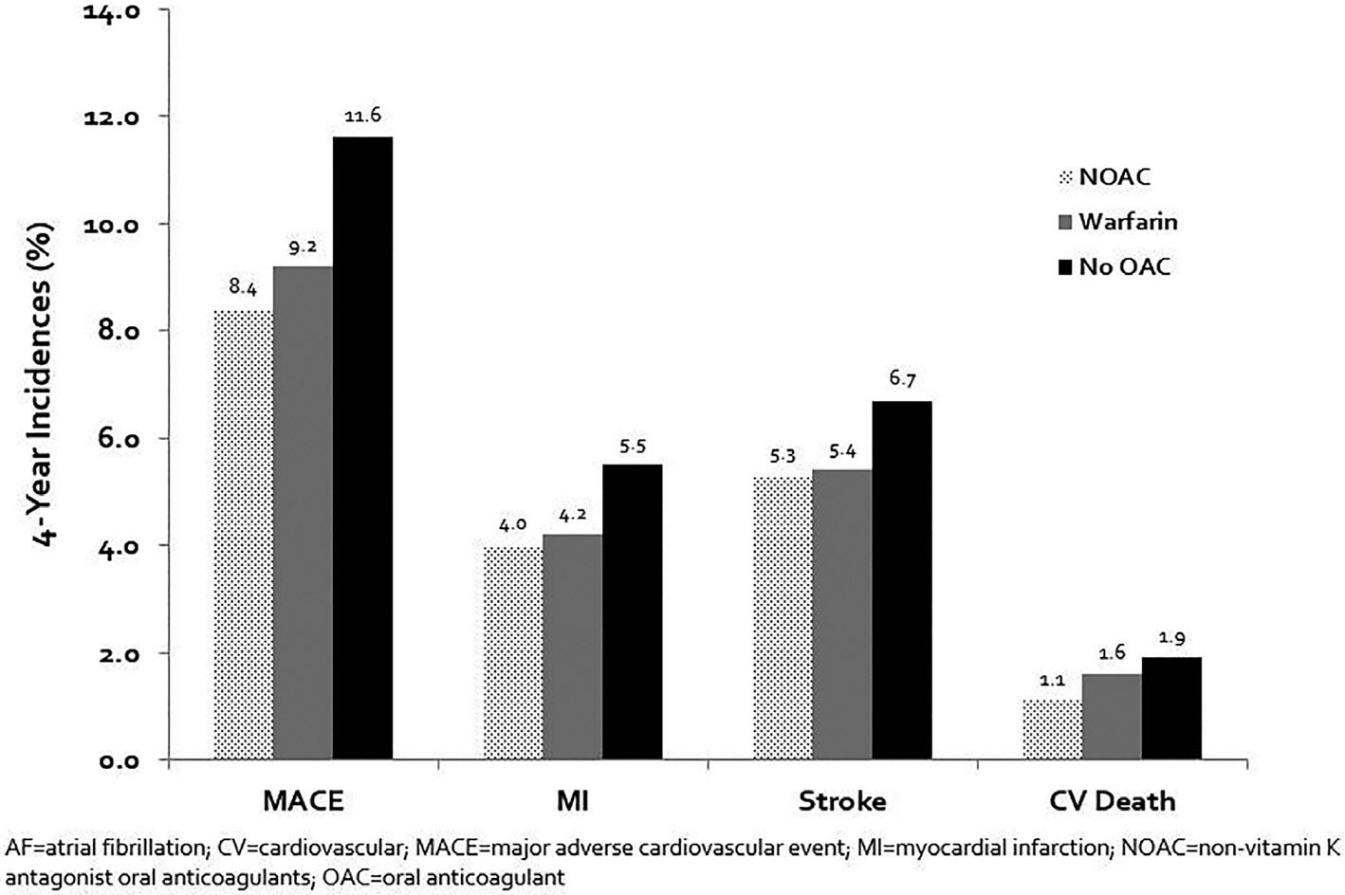
Incidence of MACE and its components according to OAC use. MACE, major adverse cardiovascular events

When the population was stratified based on CHA_2_DS_2_‐VASc scores, the incidence of MACE and its individual components increased as scores increased (*P*‐interaction <.oo01 for each outcome). MACE incidences ranged from 5.2% with a CHA_2_DS_2_‐VASc score of 0% to 19.6% with a CHA_2_DS_2_‐VASc score of 9 (Figure 2). Figure 3 shows incidences of MACE according to warfarin use, NOAC use or absence of OAC. Adjusted Cox regression analyses revealed similar results (Table 3). The presence of stage 3 or worse chronic kidney disease, diabetes and polyvascular disease were associated with a higher risk of MACE.

**Table 3:** Cox regression model of major adverse cardiovascular events at 4‐years of follow‐up

Variables	HR (95% CI)
Age (45‐64 as referent)	
65‐74	1.19 (1.09‐1.29)
75‐84	1.90 (1.75‐2.05)
≥85	3.07 (2.81‐3.35)
Female	0.98 (0.94‐1.03)
Vascular beds (RFO as referent)	
CAD only	1.21 (1.14‐1.29)
CVD only	1.60 (1.48‐1.73)
PAD only	1.13 (1.05‐1.22)
CAD + CVD	1.58 (1.40‐1.79)
CAD + PAD	1.44 (1.29‐1.59)
CVD + PAD	1.68 (1.44‐1.95)
CAD + CVD + PAD	1.87 (1.53‐2.28)
Risk factors	
Carotid stenosis	1.01 (0.95‐1.07)
CKD stage 3 or worse	1.23 (1.15‐1.31)
Congestive heart failure	1.23 (1.17‐1.30)
Diabetes	1.11 (1.05‐1.18)
Diabetic nephropathy	1.08 (0.94‐1.23)
Hypertension with treatment	1.18 (0.95‐1.46)
Hypercholesterolemia	0.94 (0.83‐1.06)
Smoker	1.35 (1.22‐1.49)
Oral anticoagulation (none as referent)	
Warfarin	0.80 (0.76‐0.85)
NOAC	0.78 (0.73‐0.84)
Medications	
ACEI or ARB	1.02 (0.97‐1.07)
β‐blocker	1.06 (1.01‐1.12)
Calcium channel blocker	1.04 (1.00‐1.09)
Diuretics	1.02 (0.97‐1.08)
P2Y12 inhibitor	1.29 (1.22‐1.37)
Statin	0.90 (0.81‐1.01)
Metformin	1.00 (0.94‐1.07)
Alpha glucosidase inhibitor	1.26 (0.84‐1.89)
DPP4 inhibitors	1.03 (0.93‐1.13)
GLP1 agonists	0.87 (0.71‐1.06)
SGLT2 inhibitors	1.73 (1.10‐2.72)
Sulphonylureas or glinides	1.07 (1.00‐1.15)
Thiazolidinediones	0.90 (0.76‐1.07)
Insulin	1.50 (1.40‐1.61)

Abbreviations: ACEI or ARB, angiotensin‐converting enzyme inhibitor or angiotensin receptor blocker; CAD, coronary artery disease; CI, confidence interval; CKD, chronic kidney disease; CVD, cerebrovascular disease; HR, hazard ratio; NOAC, non‐vitamin K antagonist oral anticoagulants; PAD, peripheral artery disease; RFO, risk factors only.

## 4 DISCUSSION

In this large, contemporary real‐world study of AF patients with or at risk for established atherosclerotic disease, MACE occurred in nearly 1 out of 10 patients at 4‐years. AF patients experienced the highest rates of MACE if there was CVD involvement followed by PAD then CAD. Higher CHA_2_DS_2_‐VASc scores were associated with increases in MACE development as well as its individual components. Compared to those that did not receive an OAC, OAC use was associated with reductions of 22%, 28%, 11%, and 14% in the incidences of MACE, myocardial infarction, ischemic stroke, and cardiovascular death, respectively.

Many of our results are consistent with those reported from the REACH registry studies, although differences do exist. The proportion of AF patients with or at risk for established atherosclerotic disease was similar: we reported 12.4% vs 10.3% reported in REACH.^3^ Incidences of MACE, however, differed. At 4‐years, 24.3% of patients in REACH experienced MACE vs 9.9% in our study. Our study found similar myocardial infarction incidence compared to those of REACH (4.6% vs 4.9%, respectively) and lower ischemic stroke rates (5.8% vs 7.7%, respectively). The reduction in stroke rates may be explained by the effectiveness of NOACs which were introduced after the completion of the REACH registry. Randomized controlled trials have shown that NOACs are at least non‐inferior and, in some cases, superior to warfarin in preventing stroke and systemic embolism in non‐valvular AF.^13^, ^14^, ^15^, ^16^ In the updated 2019 American College of Cardiology/American Heart Association/Heart Rhythm Society guideline for management of AF and in the 2019 European Society of Cardiology guidelines for the diagnosis and management of chronic coronary syndromes, NOACs are recommended over warfarin in eligible patients with non‐valvular AF (class I/level‐of‐evidence A recommendation).^5^, ^6^, ^7^ Although our study pooled NOACs and warfarin together, it captures an intermediary, real‐world population during the gradual adoption of NOACs in comparison to the REACH population which predates the approval of NOACs. In addition to these changes, several modifications to the management of AF and atherosclerotic diseases have been made since the completion of the REACH registry. These include new long‐term anticoagulation recommendations based on CHA_2_DS_2_‐VASc scores^7^ and updates to American College of Cardiology/AHA guidelines which have recommended more intensive blood pressure control, increased usage of high‐intensity statins and an increased scope and duration of treatment with dual antiplatelet therapy.^17^, ^18^, ^19^

Other studies have also evaluated the association of risk factors and AF on MACE or its components. The GARFIELD‐AF registry reported age, heart failure, prior stroke, vascular disease, moderate‐to‐severe chronic kidney disease, diabetes mellitus, and CHA_2_DS_2_‐VASc score of ≥3 to be independent predictors of higher risk of early death.^8^ Although this is generally consistent with our findings, several differences between the studies exist. The prospective GARFIELD‐AF registry focused on newly diagnosed (incident) AF patients and did not require patients to have atherosclerotic disease. Patients were also assessed for all‐cause mortality, stroke/systemic embolism at 1‐year of follow‐up, whereas our study retrospectively evaluated MACE using a cardiovascular death definition in an AF population comorbid with atherosclerotic disease over 4‐years of follow‐up. Similarly, meta‐analyses of PAD patients have found a significant association between AF and increased mortality (OR = 2.52, 95% CI = 1.91‐3.34) and diabetes and increased mortality (OR = 1.89, 95% CI = 1.51‐2.35).^20^, ^21^ The investigators also found the prevalence of AF among PAD patients to be 11.4%. This is in line with our findings in which 12.4% of patients with atherosclerotic disease had comorbid AF. Similarly, we also found diabetes to be associated with increased risk of MACE (HR = 1.11, 95% CI = 1.05‐1.18).

Usage of OAC was limited in this population. Even in patients with a CHA_2_DS_2_‐VASc score of 2 to 9 who are recommended by guidelines for anticoagulation, only 64.4% received any OAC and 20.7% received a NOAC. Physicians' fear of bleeding,^22^ especially in the context of atherosclerotic patients who are also commonly on antiplatelet agents, may help explain the lower than expected utilization of OAC in this population. However, antiplatelet therapy alone does not provide the necessary stroke prevention in patients with AF.^5^, ^6^ Randomized controlled trials have clearly demonstrated the superiority of OAC therapy over single or dual antiplatelet therapy for the prevention of stroke and systemic embolism^23^, ^24^ in AF. Compared to the REACH registry, which reported 54.2% of patients at baseline representing practice from 2003 to 2004,^3^ and to the GARFIELD‐AF registry, which had 60.4% of patients prescribed anticoagulation therapy at baseline representing practice from 2010 to 2013 (cohorts 1 and 2),^25^ OAC adoption has improved over the years potentially explaining the lower ischemic stroke rates we observed.

Another driving factor for the difference in MACE incidences is the definition of cardiovascular death between studies. Our study utilized a 14‐day grace period after a myocardial infarction, ischemic stroke, heart failure, acute coronary syndrome, coronary artery bypass grafting, or percutaneous coronary intervention for the occurrence of an in‐hospital cardiovascular‐related death. Our definition provides a conservative estimate of cardiovascular‐related deaths given the limitations of claims data. In contrast, REACH captured cardiovascular deaths outside of hospital settings. This likely explains the lower 4‐year MACE incidence in our study (1.6%) compared with REACH (10.6%).^3^

This study has limitations worth discussing. First, misclassification bias must always be considered in claims database analyses and can detrimentally affect a study's internal validity when present.^26^ Namely, our study only assessed OAC use at baseline and does not account for initiation during follow‐up. Second, clinical adjudication of events was not possible within our claims database analysis. Of note, the REACH registry, while performed prospectively, also did not independently adjudicate outcomes. Third, antiplatelet agents, such as aspirin, are acquired over the counter and are difficult to track in claims data; however, OAC therapy is superior to antiplatelet therapy and is a mainstay in AF guidelines. Fourth, we were unable to differentiate between paroxysmal and persistent AF because our baseline period in 2013 used ICD‐9 codes which do not make this distinction. Fifth, although we utilized a conservative definition of cardiovascular death which resulted in a smaller rate in comparison to that of REACH, survival bias cannot be ruled out. Finally, we used US commercial and Medicare supplemental plan claims data. As a consequence, our results are most generalizable to an insured US population with established or at high‐risk for atherosclerotic disease.^9^

## 5 CONCLUSION

In conclusion, this large, contemporary real‐world study demonstrates the impact of comorbid AF on MACE rates in patients with or at high‐risk for atherosclerotic disease. We showed that increasing CHA_2_DS_2_‐VASc scores were associated with higher MACE risk and a significant number of patients will experience a MACE by 4 years. OAC usage remains relatively low, although it may be increasing in recent years. Reductions in MACE risk may be attributed to changes in practice for AF management and the adoption of NOACs. These results emphasize the continued need for optimizing anticoagulant and antiplatelet therapy in AF patients at risk of or with atherosclerotic disease.

## CONFLICTS OF INTEREST

B.M., A.V.H., N.M., Y.M.R., and W.L.B. declare they have no conflicts of interest. C.I.C. has received research funding and honoraria from Bayer AG, Janssen Scientific Affairs LLC, and Portola Pharmaceuticals, Inc. M.J.A. reports consultancy or speaker fees and honoraria from Genentech, Janssen Pharmaceuticals, Boehringer Ingelheim, Pfizer, Bristol‐Myers Squibb, Medscape, Portola and patents/royalties from Duke University. W.L.B. has received research funding from Portola Pharmaceuticals, Inc and consultancy fees from Bayer AG.
